# PERCEPTIONS AND ATTITUDES TOWARD LONELINESS DETECTION USING PASSIVE SENSING TECHNOLOGIES IN OLDER ADULTS

**DOI:** 10.1093/geroni/igae098.2541

**Published:** 2024-12-31

**Authors:** Emma Cho, George Demiris, Hannah Cho, Insup Lee, Ahhyun Yuh, Xiayan Ji, Sean Lee Harrison, Oleg Sokolsky

**Affiliations:** University of Pennsylvania, Philadelphia, Pennsylvania, United States; University of Pennsylvania, Philadelphia, Pennsylvania, United States; University of Pennsylvania, Philadelphia, Pennsylvania, United States; University of Pennsylvania, Philadelphia, Pennsylvania, United States; University of Pennsylvania, Philadelphia, Pennsylvania, United States; University of Pennsylvania, Philadelphia, Pennsylvania, United States; University of Pennsylvania, Philadelphia, Pennsylvania, United States; University of Pennsylvania, Philadelphia, Pennsylvania, United States

## Abstract

While passive sensing has been examined in terms of older adults’ acceptance for the purpose of tracking activity levels or daily routines, less attention has been paid to how older adults perceive monitoring technologies that specifically capture levels of loneliness and social isolation. Therefore, the purpose of this study is to explore the perceptions and attitudes of older adults toward passive sensing technologies installed in their homes to assess loneliness. Structured interviews were conducted to assess older adults’ perceptions and attitudes toward passive sensing technologies to assess their loneliness after six months of having seven types of sensors (e.g, motion, temperature, bed and door sensors) installed in their homes. Audio recordings were transcribed and content analysis was conducted. A total of 15 older adults participated in the interview sessions. Themes included participants’ adaptation and adjustment, privacy and trust in data sharing, and perceived benefits and concerns of the installed sensors. Benefits included detecting and reducing loneliness, monitoring physiological parameters, improving healthier behaviors, etc. Concerns were raised about privacy, such as potential misuse by authorities or third parties, lack of accuracy of functions or feasibility of sensors, and lack of human response. All participants had an overall positive attitude towards sensors that could be installed in their homes to assess their loneliness.

